# Cognitive Enhancement Strategies for Older Adults: An Evaluation of Different Training Modalities to Improve Executive Function—A Systematic Review and Meta-Analysis

**DOI:** 10.3390/jcm13051301

**Published:** 2024-02-25

**Authors:** Sergi Rodriguez-Rodríguez, Max Canet-Vintró, Sang Ouk Wee, Jacobo Rodríguez-Sanz, Carlos López-de-Celis, Guillermo R. Oviedo, Noé Labata-Lezaun, Albert Pérez-Bellmunt

**Affiliations:** 1 Basic Sciences Department, Universitat Internacional de Catalunya, 08195 Barcelona, Spain; srodriguezr@uic.es (S.R.-R.); maxcanet44@uic.es (M.C.-V.); jrodriguezs@uic.es (J.R.-S.); goviedo@uic.es (G.R.O.); aperez@uic.es (A.P.-B.); 2 ACTIUM Functional Anatomy Research Group, Sant Cugat del Vallés, 08195 Barcelona, Spain; carlesldc@uic.es; 3 Department of Kinesiology. California State University, San Bernardino, CA 92407, USA; sangouk.wee@csusb.edu; 4 Department of Physiotherapy, Universidad de Vitoria-Gateiz (EUNEIZ), 01013 Gasteiz, Spain

**Keywords:** older adults, aerobic exercise, resistance exercise, executive function

## Abstract

(1) **Background**: The aging population is expected to triple by 2050. Executive functions decline with age, impacting daily tasks, and this is associated with neurodegenerative diseases. Aerobic and resistance exercises positively affect cognitive function in older adults by influencing growth markers. However, the modalities of exercise and the optimal parameters for maximum cognitive benefits remain unclear. (2) **Methods**: A meta-analysis of randomized clinical trials (RCTs) was conducted. The systematic search was on slowing cognitive decline and performed in the PubMed/MEDLINE and Cochrane Library databases. Articles were included if participants were ≥65 years, healthy, and performing resistance or aerobic exercise, and they were excluded if there was a combination of training and if they have neurological disease or cognitive impairment. (3) **Results**: The search strategy found a total of 1635 studies. After removing duplicates and assessing the inclusion and exclusion criteria, eight articles were included in the meta-analysis, with a total of 463 healthy older adults analyzed. No significant differences between the intervention groups and the control groups after the aerobic or resistance programs were found. (4) **Conclusions**: Aerobic exercise interventions improved executive function more than resistance training in older adults, but without statistically significant differences. This can serve as a guide to see, with caution, whether we need a multidisciplinary approach to be more effective in improving the cortical health of older adults.

## 1. Introduction

As the aging population is expected to almost triple by 2050 [1], there is a growing need for strategies to militate against common health issues associated with aging [2]. Especially age-related decline in cognitive performance has received an increment in experimental attention [3]. Cognitive decline has a negative impact on overall health, which imposes a heavy burden on families and health service systems [4].

Cognitive function refers to multiple mental abilities that include learning, thinking, reasoning, remembering, problem solving, decision making, and attention [5]. Executive function, the cognitive processes, primarily linked to the prefrontal cortex of the brain, which guide and regulate purposeful, goal-directed behavior, is one area of cognitive function that is known to decline because of the aging process [6,7]. Executive function is an umbrella for functions such as planning, working memory, inhibition, mental flexibility, and initiation and monitoring of action [8]. Because of the significance of cognitive function processes in performing daily tasks, cognitive dysfunction is associated with less functionality, which is also particularly associated with age-related neurodegenerative diseases such as Alzheimer’s [9].

From young to older adults, executive functions are associated with physical activity. The scientific literature [10,11,12] suggest that older adults use executive functions to support the execution of complex motor tasks because motor control may become less automated with age. Moreover, several studies [13,14] have shown that the older the age, the worse the score on cognitive tasks. This is relevant, as one study described that worse performance of executive functions was related to worse performance of daily living tasks [15].

To improve the decline in cognitive functions in older adults, some randomized controlled studies have shown that aerobic exercise and resistance exercise have positive effects on cognitive function in older adults [16]. It was also shown that exercise increases cerebral blood flow, reduces brain shrinkage, and improves brain tissue metabolism, leading to adaptive changes in brain function [16,17]. Previous meta-analyses [18] showed that exercise training interventions are an effective approach to improving executive function in older populations. More specifically, it has been shown that different training interventions such as resistance training and aerobic exercise improved executive function sub-domains such as working memory, cognitive flexibility, inhibition, and planning in older adults [8].

On a physiological level, exercise training stimulates growth markers that positively influence physical and cognitive function (e.g., insulin-like growth factor-1 [IGF-1]) in both the periphery and the central nervous systems [19]. As for the optimal dose, the benefits obtained depend on the intensity, frequency, type of exercise, and time, and all these parameters are still not completely clear at present [20]. As such, the precise implementation of these variables is critical to effectively elicit improvements in both physical and cognitive function [20].

Although there is a lot of literature on the effects of both aerobic and resistance exercises on physical and cognitive levels in older adults, many variables have been proposed regarding the type of exercise used, the dose, and the time. The objective of this review is to evaluate, through an analysis of the scientific literature, which exercise modality is most optimal to improve the executive functions of older adults.

## 2. Materials and Methods

This systematic review and meta-analysis were performed in accordance with the Preferred Reporting Items for Systematic Reviews and Meta-Analyses (PRISMA) statement [21]. The protocol of this systematic review was published in the International Prospective Register of Systematic Reviews (PROSPERO: CRD42023467344).

### 2.1. Systematic Literature Search

We conducted a systematic literature search for studies included up to July 2023 in the electronic databases PubMed and Cochrane. Four domains (participants, intervention, outcomes, and study design) were used for the search strategy. The main terms selected were (1) participants: elderly OR older adults; (2) intervention: aerobic exercise OR aerobic training OR resistance training OR resistance exercise; (3) outcomes: cognitive functions OR executive functions OR cognitive abilities; and (4) study design: the search strategy proposed was used to include only RCT. The complete search strategies are available in the Supplementary Material S1.

### 2.2. Selection Criteria

The systematic review and meta-analysis included RCT with a sample of older adults ≥ 65 years old without cognitive impairment. Studies had to use as intervention, aerobic/cardiovascular (AE) or resistance exercise (RE). At least one of the following outcomes had to be reported in the primary articles to be included: cognitive function, executive function, or cognitive skills. We excluded articles that (1) had participants with neurological disease or cognitive impairment; (2) had interventions combining both AE and RE or other interventions (balance and pulmonary rehabilitation); and (3) were written in languages other than English. We also excluded review articles, book chapters, and study protocols.

### 2.3. Screening, Selection Process, and Data Extraction

After removing duplicate papers, the articles were independently screened by 2 reviewers (S.R.R. and N.S.A.) to identify appropriate articles by the titles and abstracts. We used Rayyan.ai software (https://rayyan.qcri.org, accessed on 15 August 2023) [22] to eliminate duplicate articles and for identifying relevant works by titles and abstracts. The criteria used by the two reviewers during the initial screening involved including articles only if the titles and abstracts were related to the topic studied and the specific population of older adults. Both reviewers (S.R.R. and N.S.A.) used a standardized form to independently extract data from each paper, where they included the following: author’s last name, year of publication, study design, type of intervention, comparison, study variables, and results.

### 2.4. Assessment of Methodological Quality and Risk of Bias

Risk of bias and methodological quality of the included trials were independently assessed by 2 authors (S.R.R. and N.L.L.) using the Cochrane Risk of Bias (RoB2), a widely accepted tool to evaluate the quality of an RCT in the biomedical field, proposed by the Cochrane Collaboration [23] and the Physiotherapy Evidence Database (PEDro) scale [24]. These are especially designed to assess the quality of physiotherapy trials based on random allocation, concealed allocation, baseline between-group similarity, blinding of participants, therapists and assessors, dropouts, intention-to-treat statistical analysis, between-group statistical comparison, point measures, and variability data [24]. Both authors (S.R.R. and N.L.L.) considered the combination of these 2 tools to increase the reliability of the assessment of the methodological quality.

### 2.5. Data Synthesis and Analysis

Review Manager 5 (Cochrane Collaboration, Oxford, UK) was used for all statistical analyses. If data were not reported in the paper, the authors were contacted by e-mail to obtain the necessary data to be included in the quantitative analysis. In this systematic review and meta-analysis, the mean difference was chosen as the effect size when studies used the same tool of measure. The standard mean difference (SMD) was chosen as the effect size when studies used different tools to measure the outcome. A 95% confidence Interval (CI) for all the effect sizes was used. The inverse of the variance (IV) statistical test was used for the quantitative analysis. In this systematic review and meta-analysis, a random effects model was used to determine the overall effect size because the number of included studies was small [25]. We considered *p* < 0.05 as statistically significant. Heterogeneity was calculated by the I^2^ statistic, being classified as low (I^2^ ≤ 25%), moderate (25 < I^2^ < 50%), or high (I^2^ ≥ 50%) [26].

## 3. Results

**Figure 1:**
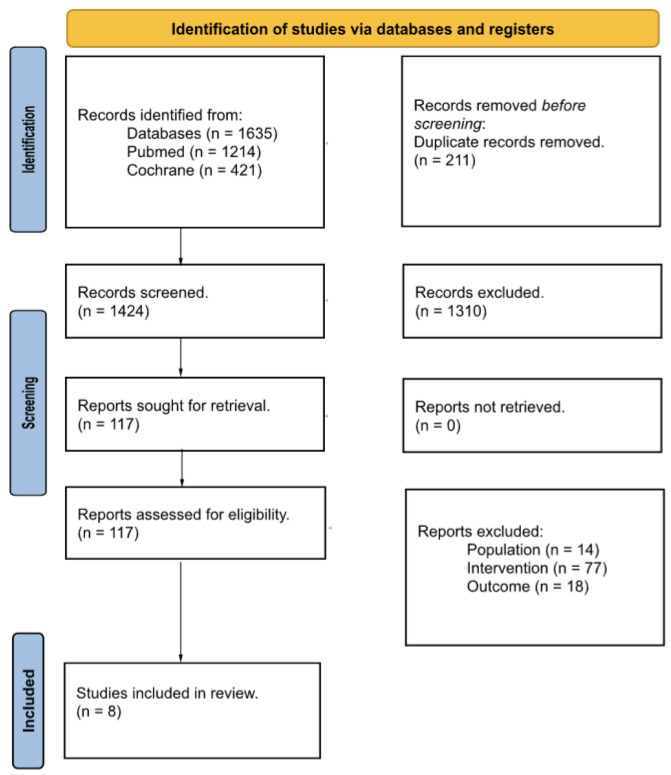
PRISMA 2020 flow diagram.

The search strategy found 1635 studies (PubMed: 1214 and Cochrane Library: 421). After removing duplicates, a total of 1424 studies were initially considered to be included in the study. After screening the titles and abstracts for not meeting the inclusion criteria, 1310 studies were excluded. A total of 114 studies remained in the study, and after assessing the full text, 8 studies met the inclusion criteria for the quantitative analysis, and 109 studies were rejected because the population, sample, or outcomes were not those studied in this study (Figure 1). One study was dropped from the meta-analysis for not presenting the required data after the authors had been contacted.

### 3.1. Study Characteristics

In Table 1, we summarize the sample characteristics of all the articles included [27,28,29,30,31,32,33,34]. A total of 463 older adults, each over 65 years old, were included in this systematic review. We saw that the distribution was greater for women, which was 72,2% of the study population. The sample size varied between 16 [27] and 119 [28] individuals, with an average age between 65.8 [29] and 75.2 [28] years old. Regarding the types of the interventions, five studies included aerobic training in the experimental group, and four studies included resistance training in the experimental group. Only one study (Iuliano et al. 2015) included both interventions, aerobic training and resistance training. For the control group, six studies included a non-training group, one included an educational session [28], and another one included stretching exercises [30]. Finally, regarding the study variables, executive function was assessed with the Stroop Color Test in four studies [29,30,33,34], with the Stroop Task Performance in one study [27], with the Mini Mental State Exam in one study [31], with the Task Switching Test in one study [28], and with the Wisconsin Card Sorting Test (WCST) in one study [32].

### 3.2. PEDro Score

The average score for the PEDro scale can be found in Table 2. The score was 5/10, and was considered a “fair” methodological quality across the studies included in the systematic review. When we analyzed the main methodological problems, we found that 0/8 studies did not have their participants blinded, and 0/8 studies did not have their therapists blinded.

### 3.3. Risk of Bias Graph and Summary

**Figure 2:**
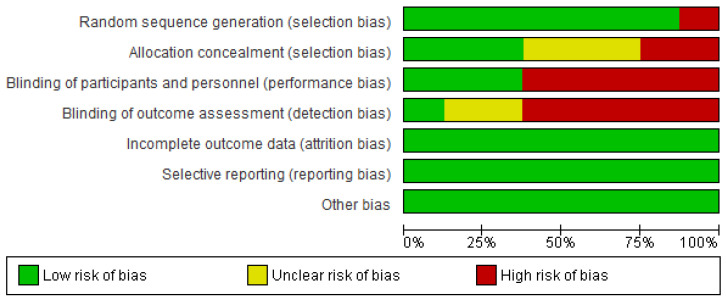

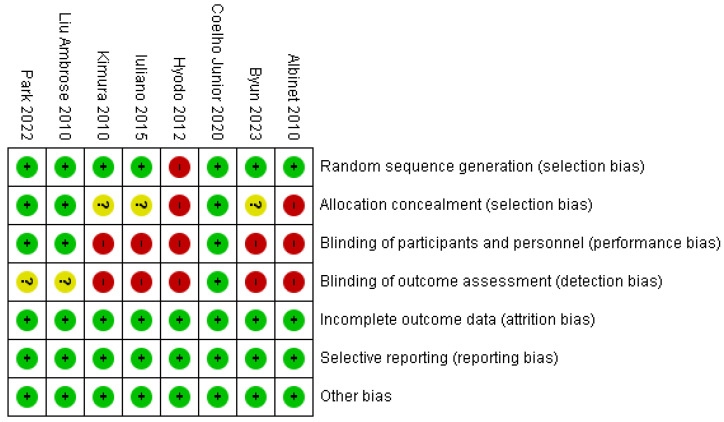
Risk of bias graph and summary [27,28,29,30,31,32,33,34].

Figure 2 shows the Rob 2 tool graph and summary. We found a risk of bias in terms of performance bias (5/8) and detection bias (5/8). We found a low risk of bias in terms of selection bias (7/8) and attrition bias, reporting bias, and other biases (8/8). (+) signs in green indicate a low risk of bias; (?) signs in yellow indicate unclear a risk of bias, and red marks indicate a high risk of bias.

### 3.4. Effectiveness of Interventions

#### 3.4.1. Resistance Exercise

**Figure 3:**
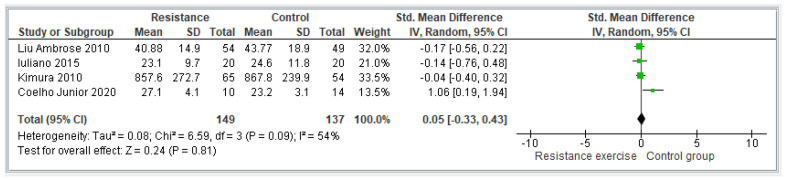
Effectiveness of resistance exercise on executive function [28,29,30,31].

Four articles [28,29,30,31] reported results about resistance exercise. Regarding all the studies, the study by Liu Ambrose et al. [30] focused on resistance training. They found, among their results, improvements in the Stroop performance compared with the control group. In the study conducted by Iuliano [29], the authors focused on high-intensity strength training, and no significant improvements were found between pre- and post-measures of the experimental group. In the study conducted by Kimura et al. [28], the authors focused on progressive resistance training. They found significant changes between pre- and post-intervention executive functions. Finally, for the study conducted by Coelho Junior et al. [31], the authors focused on resistance training, and found significant cognitive function improvements in comparison with the control group. Considering the four studies that focused on resistance exercise, there was a total sample of 286 participants. Figure 3 shows the comparison between resistance training and the control group. An analysis shows an overall SMD of 0.0 [ 95%CI −0.33, 0.43] and an overall effect of Z= 0.24 (*p* = 0.81). The heterogeneity was considered moderate (I^2^ = 54%).

#### 3.4.2. Aerobic Exercise

**Figure 4:**
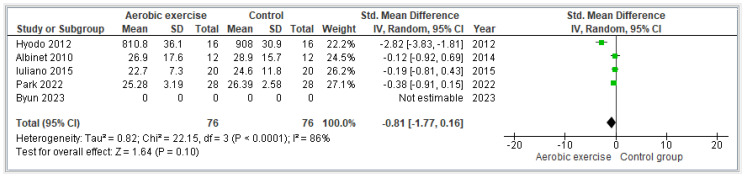
Effectiveness of aerobic exercise on executive function [27,29,32,33,34].

Four articles [27,29,32,33,34] reported results about aerobic exercise. The only study that could not be incorporated into the meta-analysis was the Byun et al. study [33], because after requesting the data from the authors, no response was received, and the data could not be incorporated. The study conducted by Hyodo et al. [27] focused on an acute bout of moderate aerobic exercise. They found significant improvements in cognitive performance after the intervention program. In their study, Albinet et al. [32] focused on an aerobic training program to enhance aerobic endurance. They also found significant results in the aerobic group in comparison with the control group. On the other hand, for the study conducted by Iuliano et al. [29], the authors focused on a high-intensity cardiovascular training. They found no significant differences in the scores of the cognitive functions after the intervention for the aerobic exercise group. Finally, in the study conducted by Park et al. [34], the authors focused on aerobic stepping exercise. They found greater improvements in executive function than those in the control group. Considering the four studies, there was a total sample of 152 participants. Figure 4 shows an overall SMD of −0.81 [−1.77, −0.16] and an overall effect of Z = 1.64 (*p* = 0.10). The heterogeneity was considered high (I^2^ = 86%).

## 4. Discussion

The objective of the study is to analyze the effects of two different types of exercise (AE or RE) on the executive function in older adults. This meta-analysis summarizes results from a total of eight studies and 463 participants, and shows the results obtained. After describing the results in the previous section, this systematic review and meta-analysis show no significant statistical differences in both aerobic and resistance exercises and their respective control groups in terms of improving executive function in older adults. Possible causes of this non-statistically significant result could include inadequate methodology, heterogeneity, and a small sample size.

### 4.1. Resistance Exercise

It is important to note that the heterogeneity between studies was moderate for resistance exercise (I^2^ = 54%). This percentage may be due to the difference in the years of publication between the different articles included. Furthermore, it may also be due to the difference in the sample size of the different articles included.

The result of our meta-analysis shows no significant differences between the resistance exercise and the control groups in terms of improving executive function in older adults. These findings differ from another study [35], where the authors showed significant interaction effects for resistance training with executive function, suggesting that this type of training has particularly pronounced effects on these domains of cognitive function. On the other hand, our results are similar to those of other studies [36,37,38,39], where they fail to show any consistent evidence for the benefits of resistance training on different domains of cognitive function.

First, the differences observed may have been because the studies did not use the same executive function measurements or scales and needed to include another measurement for the executive function. In the second place, they may be explained by the duration of the interventions. In this metanalysis, the articles that used resistance exercise as the type of intervention had an intervention duration of less than 6 months, except the study of Coelho-Junior et al. [31]. The duration of these interventions is a critical factor because exercise interventions of less than 6 months have demonstrated controversial findings on cortical function [40]. In fact, Gomes-Osman et al. [41] showed a meaningful relationship between the total length of target exercise interventions and cognitive gains in older adults with and without cognitive impairment, suggesting a potential “length threshold” to boost brain health.

### 4.2. Aerobic Exercise

For this type of intervention, it is important to highlight that heterogeneity was high (I^2^ = 86%). This may have occurred, firstly, due to the difference in year of publication and secondly, due to the difference in the sample and the timing of the interventions.

The results of our study show that the aerobic exercise group had better cognitive performance after the intervention, but the results were not statistically significant (*p* = 0.10) in comparison with the control groups. The present results are in line with the following study by Frost et al. [42], where they also found no statistically significant group effect on executive functions after a cycling program. This is because, as different studies have explained [43,44], aerobic exercise alone is not enough to produce significant changes in the executive function. Another factor that can explain our results is the duration of the interventions, taking into account that in our reviewed studies, Albinet et al. [32] carried out 12 weeks of intervention and Park et al. [34] carried out 5 weeks; perhaps a greater weekly duration of exercise is required to induce cognitive health [42]. Moreover, Coetsee et al. [44] also found that the accuracy on the two Stroop tasks did not change significantly after their interventions in any of the aerobic groups (moderate and high-intensity). These results relate to those of our study and could be due, as described by Kramer et al. [45] to the fact that aerobic exercise has a selective effect on cognition, without significantly modifying parameters of executive function.

On the other hand, the study by Smith et al. [46] differs from our results, after reviewing nineteen studies and obtaining significant results (*p* = 0.018) in their study in attention and processing speed, executive function, and memory. In the present study, it may have been the case that because the sample of studies analyzed with aerobic exercise was so small, we did not find statistically significant results with respect to executive function, even though we observed in the studies analyzed that there were positive changes in executive function after the interventions.

### 4.3. Need for New Therapeutic Strategies

Therapeutic strategies are an important factor to take into account when carrying out different exercise modalities in older adults and can explain our results of the program’s personalization [47].

The interplay between an individual’s intrinsic characteristics, behaviors, and environmental/ecological influences is crucial to achieving the optimal trajectory [47]. In our study, the fact of not having considered the intrinsic capacities of each individual to personalize the exercise may have been a factor in the results not being significant. As Gronwald et al. [48] said in their article, exercise prescriptions should be individualized, adjusted, and controlled like any other medical treatment.

Another critical factor is the type of therapeutic approach. As Kim et al. describe in their study [49], despite the importance of exercise therapy, some older adults tend to refuse exercise itself due to their malfunctioning body, which means that we need to devise new therapeutic approaches that achieve similar results. This is a relevant factor, because it could be that in the articles included in our study, many of the participants who carried out the aerobic or resistance exercise programs could not achieve the expected benefits due to their abilities, motivations or limitations, and another therapeutic approach would have been a better option to improve cognitive function. 

A possible future line, as Heesterbeek et al. [50] describe in their study, to improve cognitive functioning in older adults with cognitive decline, can be body vibration exercise programs. They found that the vibratory stimulus with exercise can be a promising intervention, as it induces brain activation in the sensory cortex and increases blood circulation safely, influencing cognitive function positively through activated cerebrovascular circulation [49,50]. It could be a promising therapeutic tool for those older adults who cannot perform an aerobic or resistance exercise protocol due to their intrinsic abilities and motivational factors or because they are in a frail situation.

### 4.4. Limitations of the Study

It is important to mention the heterogeneity of the studies, as well as the intrinsic characteristics of the studies. These differences are mainly related to the number of participants, the type of interventions, and the duration of the interventions. Another consideration is that the articles analyzed on resistance training were evaluated with the MMSE scale, which is less sensitive to changes that may occur. Moreover, the small number of studies analyzed becomes another limitation. One more study constraint was not being able to obtain the data from the author of one of the studies and to add them to the meta-analysis.

## 5. Conclusions

After analyzing our results, we can conclude that aerobic exercise is a training modality that produces better changes regarding executive function compared to resistance exercise. Even so, the positive changes observed with aerobic exercise are not statistically significant. This can serve as a guide to see, with caution, the importance of aerobic exercise at the cognitive level and continue investigating whether we need a different approach to be more effective in improving the cortical health of older adults.

More clinical trials with a larger number of studies should be carried out to continue investigating the effects of exercise on a cognitive level and to investigate what types of interventions are most effective in improving the health of older adults.

## Figures and Tables

**Table 1:** Study characteristics.

Study	N (IG/CG)	Age (±SD)	Gender (F/M)	Modality	Variable
Albinet et al., 2010 [32]	24 (12/12)	70.7 ± 4.2	13/11	AE	WCST
Byun et al., 2023 [33]	81 (40/41)	68.2	61/20	AE	SCT
Coelho Junior et al., 2020 [31]	24 (10/14)	67.0 ± 6.2/66.7 ± 4.8 *	24/0	RE	MMSE
Hyodo et al., 2012 [27]	16 (16/16)	69.3 ± 3.5	3/13	AE	ST
Iuliano et al., 2015 [29]	40 (20/20)	65.8 ± 6.3/66.4 ± 6.3 *	23/17	RE	SCT
Iuliano et al., 2015 [29]	40 (20/20)	68.4 ± 6.4/66.4 ± 6.3 *	24/16	AE	SCT
Kimura et al., 2010 [28]	119 (65/54)	73.6 ± 4.7/75.2 ± 6.3 *	70/39	RE	TST
Liu Ambrose et al., 2010 [30]	103 (54/49)	69.6 ± 3	155/0	RE	SCT
Park et al., 2022 [34]	56 (28/28)	67.7 ± 4.1/67.7 ± 4.9 *	28/28	AE	SCT

IG: intervention group; CG: control group; RE: resistance exercise; AE: aerobic exercise; M: male; F: female; SD: standard deviation; ST: Stroop Task; SCT: Stroop Color Test; MMSE: Mini Mental State Exam; TST: Task Switching Test: WCST: Wisconsin Card Sorting Test. * Intervention Group/Control group.

**Table 2:** PEDro scale.

Author	1	2	3	4	5	6	7	8	9	10	11	Total
Albinet et al., 2010 [32]	0	1	0	1	0	0	0	0	0	1	1	4
Byun et al., 2023 [33]	1	1	1	1	0	0	1	1	0	1	1	7
Coelho Junior et al., 2020 [31]	1	0	0	1	0	0	0	0	0	1	1	3
Hyodo et al., 2012 [27]	1	1	0	1	0	0	0	1	0	1	1	5
Park et al. [34]	1	1	1	1	0	0	1	1	1	1	1	8
Iuliano et al., 2015 [29]	1	1	0	1	0	0	0	0	1	1	1	5
Kimura et al., 2010 [28]	1	1	1	1	0	0	0	0	0	1	1	5
Liu Ambrose et al., 2010 [30]	1	1	0	1	0	0	0	0	0	1	1	4
Average												5 *

1: Inclusion/exclusion criteria; 2: random allocation of participants; 3: concealed allocation; 4: similarity between groups at baseline; 5: participant blinding; 6: therapist blinding; 7: assessor blinding; 8: fewer than 15% dropouts; 9: intention-to-treat analysis; 10: between-group statistical comparisons; 11: point measures and variability data. * Item 1 was not used to calculate the PEDro score.

## Data Availability

Data are contained within the article.

## Supplementary Materials

The following supporting information can be downloaded at: https://www.mdpi.com/article/10.3390/jcm13051301/s1.
