# Supplementary material for: Cognitive Enhancement Strategies for Older Adults: An Evaluation of Different Training Modalities to Improve Executive Function—A Systematic Review and Meta-Analysis
Source: J Clin Med. 2024 Feb 25;13(5):1301. doi: 10.3390/jcm13051301 (PMC10931602; doi:10.3390/jcm13051301)
Supplement: Supplementary file 1 [file jcm-13-01301-s001.zip › jcm-2887486-supplementary.pdf]

Supplementary Material S1a. Keywords used for the search strategy.

Population	Intervention	Outcomes
Older adults<br>Aged<br>Old people<br>Elderly<br>Aging<br>Older people<br>Old adults<br>Seniors	Resistance training<br>Resistance exercise<br>Strength training<br>Aerobic training<br>Aerobic exercise<br>Cardiovascular exercise	Executive function

Supplementary Material S1b: Complete literature search.

<u>PubMed Search Formula</u>

Date: 19-06-2023

(older adults OR "old people" [Title] OR "older people" [Title] OR "older adults" [Title] OR "old adults" [Title] OR elderly OR senior [Title] OR Aged [Title]) AND ("randomized controlled trial"[Publication Type] OR "controlled clinical trial"[Publication Type] OR "randomized"[Title/Abstract] OR "placebo"[Title/Abstract] OR "clinical trials as topic"[MeSH Terms] OR "randomly"[Title/Abstract] OR "trial"[Title]) AND (Executive function) AND ("aerobic exercise" OR "aerobic training" OR “cardiovascular exercise” OR "resistance training" OR "resistance exercise" OR “strength training”)

<u>Cochrane Library Search Formula</u>

Date: 26-06-2023

(older adults OR "old people" OR "older people" OR "older adults" OR "old adults" OR “elderly” OR “senior” OR “Aged”) in Title Abstract Keyword AND ("aerobic exercise" OR "aerobic training" OR “cardiovascular exercise” OR "resistance training" OR "strength training" OR “resistance exercise”) in Title Abstract Keyword AND (Executive function)
